# Preparation and Electrothermal Transport Behavior of Sn_8_[(Ga_2_Te_3_)_34_(SnTe)_66_]_92_ Bulk Glass

**DOI:** 10.3390/ma17194809

**Published:** 2024-09-30

**Authors:** Yaqi Zhang, Feng Guo, Huan Zhang, Mingming Zhang, Jianxiu Su, Zhengxin Li

**Affiliations:** 1 Postdoctoral Research Base, School of Mechanical and Electrical Engineering, Henan Institute of Science and Technology, Xinxiang 453003, China; zhangyaqi_1988@163.com (Y.Z.); guofeng200012@163.com (F.G.); mmzhang@hist.edu.cn (M.Z.); dlutsu2004@126.com (J.S.); 2 School of Mechanical and Electrical Engineering, Henan University of Technology, Zhengzhou 450001, China; zhengxin_li@haut.edu.cn; 3 State Key Laboratory of Metastable Materials Science and Technology, College of Materials Science and Engineering, Yanshan University, Qinhuangdao 066004, China

**Keywords:** glass transition, tellurium-based glasses, spark plasma sintering, electrical conductivity, thermal conductivity

## Abstract

High-conductivity tellurium-based glasses were anticipated to be the attractive candidates in chalcogenide glass systems on account of their distinctive characteristics and extensive application prospects. In this paper, the high-density (>96%) Sn_8_[(Ga_2_Te_3_)_34_(SnTe)_66_]_92_ bulk glass with the density of 5.5917 g/cm^3^ was successfully prepared by spark plasma sintering (SPS) technology at 460 K, using a 5 min dwell time and 450 MPa pressure. The room-temperature thermal conductivity of Sn8 bulk materials significantly decreased from 1.476 W m^−1^∙K^−1^ in the crystalline sample to 0.179 W m^−1^∙K^−1^ in the glass, and the Seebeck coefficient obviously increased from 35 μV∙K^−1^ in to 286 μV∙K^−1^, indicating that the glass transition of tellurium-based semiconductors could optimize the thermal conductivity and Seebeck coefficient of the materials. Compared to the conventional tellurium-based glassy systems, the fabricated Sn8 bulk glass presented a high room-temperature conductivity (*σ =* 6.2 S∙m^−1^) and a large glass transition temperature (*T_g_ =* 488 K), which was expected to be a promising thermoelectric material.

## 1. Introduction

Glassy materials have been attracting widespread interest due to their unique structures and superior physical and mechanical properties [1,2,3,4,5,6,7]. Tellurium-based glasses exhibited the infrared cut-off edges up to 25 µm, rendering them essential materials for far-infrared detection and spectral identification and being extensively employed for the exploration of life in space and the monitoring of Earth-like planets, such as the optical system of the European Space Agency’s Darwin project [8,9,10,11]. Additionally, tellurium-based glasses have also been applied in military and civil applications such as precision guidance, night-vision targeting, and security monitoring owing to their excellent optical properties in terms of fast nonlinear optical response and high nonlinear refractive index [12,13,14]. Recently, tellurium-based glasses were characterized by larger atomic weights and lower chemical bonding constants than traditional inorganic [15,16], bulk metallic and semiconductor glasses (e.g., sulfur-based and selenium-based glasses), which significantly degrade the phonon energy and transmission rate, presenting lower thermal conductivities and higher Seebeck coefficients, making tellurium-based glasses appear as newly emerging thermoelectric materials, which were of great value in mitigating the energy crisis and improving the recovery of waste heat [17,18,19,20,21]. It is also notable that the higher the electrical conductivity of tellurium-based glasses, the superior the thermoelectric performances. Meanwhile, the high electrical conductivity can further strengthen the driving force of the electric field and improve the efficiency of fingerprint identification and greenhouse effect detection, which were equally significant in the field of bio-sensing detection [22,23]. Therefore, high-conductivity tellurium-based glasses became the attractive subjects in chalcogenide glass systems owing to their extensive application prospects.

Recent studies have revealed that Sn_8_[(Ga_2_Te_3_)_34_(SnTe)_66_]_92_ tellurium-based glass presented the narrow band-gap and high glass transition temperature [24], which was anticipated to be one of the high-conductivity tellurium-based glass with potential for thermoelectric applications. However, the Sn8 amorphous ribbon samples prepared by the melt spin-dumping technique resulted in poor reliability of some experimental results and hindered applications due to the limited dimensions. Therefore, the investigations of Sn8 bulk glass samples were of greater practical significance. Spark plasma sintering (SPS) technology has become the preferred method for preparation of bulk glass owing to its advantages, such as fast heating rate, low sintering temperature, short time, and high sample densities [25,26]. For instance, in Zr-based and Fe-based glass systems, Chang and Paul discovered that the amorphous powder particles would generate spark discharge and form plasma during the energization process [27,28]. With the increase in sintering pressure, the plasma collides with the powder particles, resulting in the increase insurface activity, the decrease of the particle gaps, and the enhancement of the diffusion of the material, which lead to the increase in densification and eventually forming the high-density bulk glassy samples.

Therefore, the research of high-conductivity bulk tellurium-based glasses would be of wider application prospects and practical significance. In this paper, the preparation and electrothermal transport behavior of Sn_8_[(Ga_2_Te_3_)_34_(SnTe)_66_]_92_ bulk glass samples have been systematically investigated. Firstly, the Sn8 amorphous ribbons prepared by melt spin-dumping were placed into the agate mortar and ground to micron-scale glassy powders for the subsequent sintering. Then, the spark plasma sintering (SPS) technique was applied to realize the preparation of high-density bulk glass samples, and the parameters of sintering pressure and temperature were systematically investigated. The high-density (>96%) Sn_8_[(Ga_2_Te_3_)_34_(SnTe)_66_]_92_ bulk glass was successfully prepared by the SPS at 460 K, using a 5 min dwell time and 450 MPa pressure. Finally, the electrical conductivity, thermal conductivity, and Seebeck coefficient Sn_8_[(Ga_2_Te_3_)_34_(SnTe)_66_]_92_ bulk glass samples and crystalline samples were measured by utilizing ZEM-3 and a laser thermal conductivity meter (TC-9000). The glass transition of tellurium-based semiconductors significantly optimized the thermal conductivity and Seebeck coefficient of the materials. Meanwhile, the fabricated Sn_8_[(Ga_2_Te_3_)_34_(SnTe)_66_]_92_ bulk glass also presented a high room-temperature conductivity and a large glass transition temperature, revealing promising prospects for thermoelectric applications.

## 2. Experimental Methods

High-purity metals and compounds of Sn (99.99%, Aladdin, Shanghai, China, CAS:7440-31-5), SnTe (99.9%, Aladdin, Shanghai, China, CAS:12040-02-7) and Ga_2_Te_3_ (99.9%, Alfa Aesar, Shanghai, China, CAS:12024-27-0) were weighed on the ratio of Sn_8_[(Ga_2_Te_3_)_34_(SnTe)_66_]_92_, denoted as Sn8. The Sn8 crystalline ingots were obtained by the melt quench–cooling method, similar to the preparation of bulk metallic glasses or high-entropy alloys [29,30]. The Sn8 amorphous powder samples were prepared by melt spin-dumping techniques, as detailed in previous studies [24]. The obtained crystalline ingots and amorphous powder were ground into micron-scale powders for the subsequent spark plasma sintering (SPS). Notably, the cemented carbide mold with higher pressure-bearing capacity (500 MPa) was applied to investigate the preparation of Sn8 bulk samples as well as the influence of the sintering parameters on the density and microstructure of the samples. The density (*ρ*) was determined by the Archimedes method in ethanol (*ρ =* 0.7893 g/cm^3^) and measured three times for each sample, and the results were averaged and recorded as the final density value. The theoretical density of the Sn8 sample was estimated from reference to the densified crystalline material sintered in the cubic press machine under a high pressure at 5 GPa and at 800 K for 2 h, and the resulting value was 5.8126 g/cm^3^. The specific heats (*C_p_*) were characterized by Differential Scanning Calorimetry (DSC-8000), manufactured by Perkin-Elmer, Connecticut, USA. Based on the rule of the “three-line method”, the heat capacity curves of the sample, the sapphire, and the empty disk were sequentially measured, and the *C_p_* value was finally calculated by difference calculation. The structures were characterized by X-ray diffraction (XRD, Rigaku, Cu *K*_α1_ radiation, λ = 1.5406 Å) and transmission electron microscope (TEM, JEOL JEM-2010, Tokyo, Japan ).

**Figure 1:**
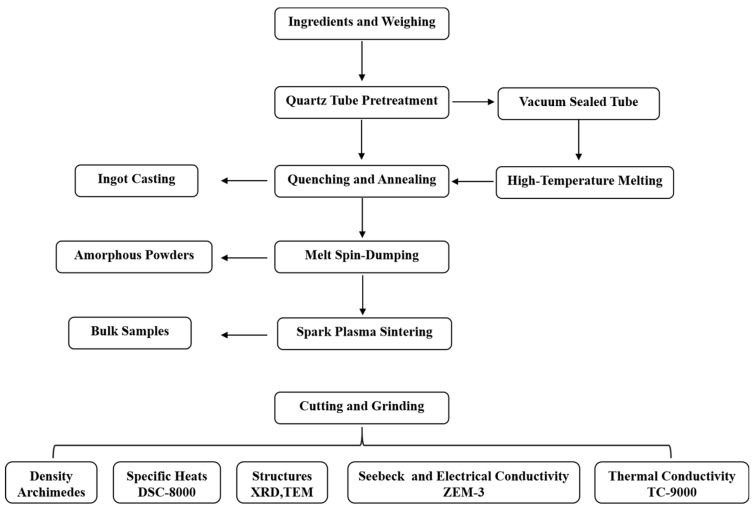
Diagram of sample preparation and characterization.

The Seebeck coefficient and electrical conductivity of the samples (2 mm × 2 mm × 6 mm) were measured based on the standard four-probe method manufactured by ZEM-3 (Ulvac-Riko Inc., Tokyo, Japan). The thermal conductivity was calculated by *κ = C_p_* × *ρ* × *D*, where the thermal diffusivity coefficient *D* was measured by the laser thermal conductivity device (TC-9000, Ulvac-Riko Inc., Japan). More than 10 consecutive measurements of electrical transports and thermal properties were carried out at different fixed temperatures within the tested temperature range, as detailed in previous studies [18]. For both glassy samples and crystalline ingots, the electrical conductivity, Seebeck coefficient, and thermal diffusivity coefficient only have a little amount of data fluctuation, exhibiting good stability and replicability. The measurement errors for electrical conductivity, Seebeck coefficient, and thermal conductivity were approximately ±3%, ±5%, and 5%, respectively. The diagram of sample preparation and characterization as shown in Figure 1.

## 3. Results and Discussions

### 3.1. Sn8 Bulk Glass Preparation

**Figure 2:**
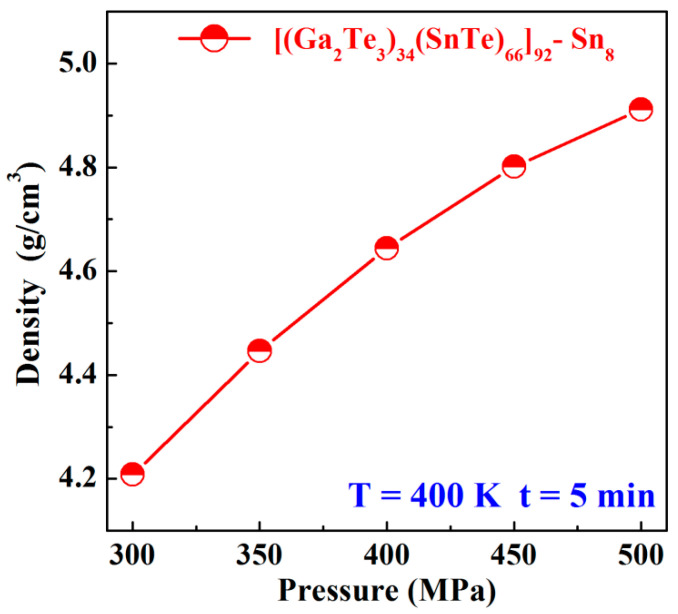
Density varied with the sintering pressure under the sintering temperature at 400 K and 5 min dwell time for Sn8 samples.

The density varied with the sintering pressure under a sintering temperature of 400 K and with a 5 min dwell time for Sn8 samples, which are shown in Figure 2. The density of the sample increased significantly from 4.2083 g/cm^3^ to 4.9116 g/cm^3^ as the pressure went up from 300 MPa to 500 MPa. This was attributed to the dramatic reduction of pores among the powder particles as the sintering pressure increased, which promoted the movements and diffusions of atoms, resulting in the denser structure of the samples. The similar experimental phenomenon was also confirmed in the Zr-Al-Ni-Cu bulk glass system [31]. The increasing trend of the density gradually became slower as the sintering pressure reached 450 MPa, and when the maximum pressure limit of the cemented carbide mold was increased to 500 MPa, the density of the sample was 4.9116 g/cm^3^, accounting for only 84.4% of the theoretical density (5.8126 g/cm^3^). This was due to the poor plasticity and viscous flow of the amorphous samples under the lower sintering temperature of 400 K, which rendered it difficult to attain the highly densification. 

**Figure 3:**
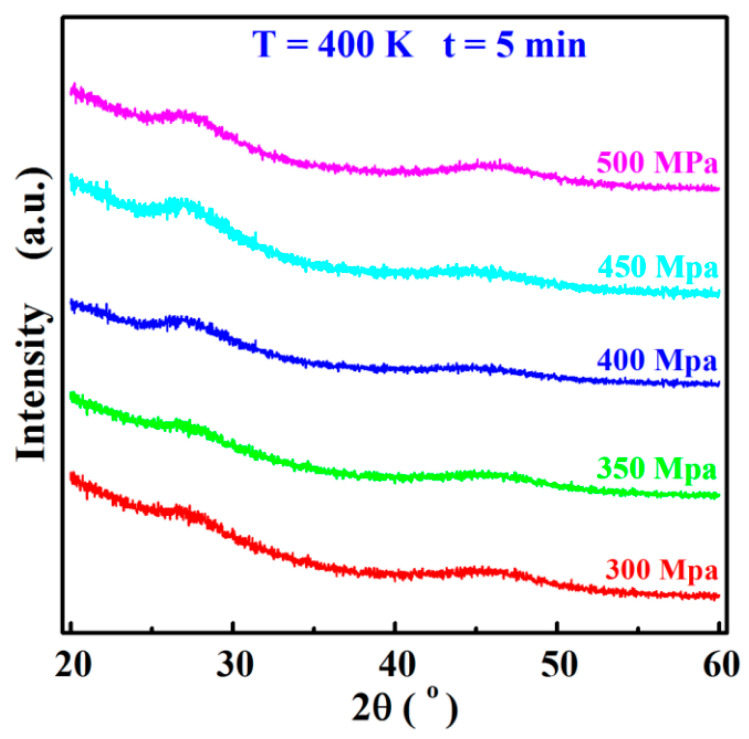
XRD patterns of Sn8 samples with different sintering pressure.

Figure 3 illustrates the XRD patterns of Sn8 samples with different sintering pressures. The diffraction patterns of all the samples exhibited the broadening amorphous diffuse scattering peaks without identifying any crystalline characteristic peaks, demonstrating that all the sintered samples remained in the completely glassy structure under the mentioned sintering parameters. In order to guarantee that the sintered samples possessed the relatively high density while preventing the molds from being shattered during the fabrication, the sintering pressure of the Sn8 samples was finally determined to be 450 MPa.

**Figure 4:**
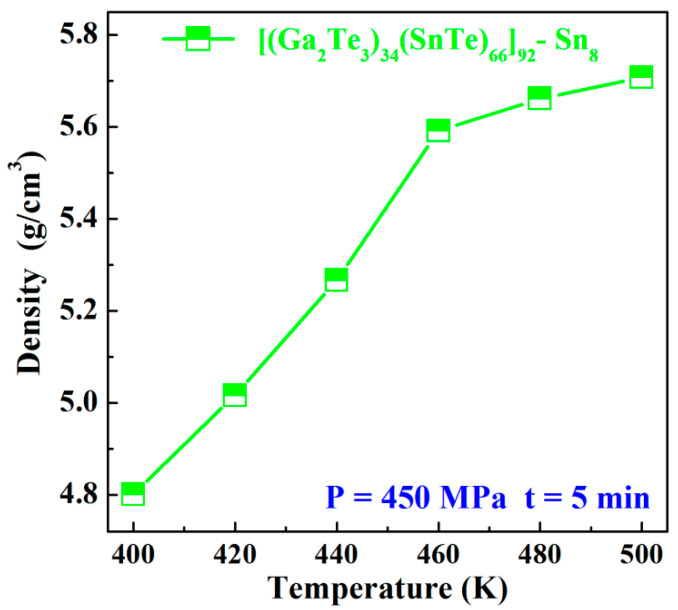
Density varied with the sintering temperature under the sintering pressure at 450 MPa and 5 min dwell time for Sn8 samples.

Figure 4 presents the variation of density as a function of sintering temperature under the sintering pressure at 450 MPa and 5 min dwell time for Sn8 samples. The density of the samples displayed a tendency of rapidly increasing and then slowly increasing as the sintering temperature rose. In particular, a significant growth in the density from 5.0163 g/cm^3^ to 5.5917 g/cm^3^ was detected when the sintering temperature was increased from 420 K to 460 K, and the densification increased from 86.3% to 96.2%. On the one hand, the diffusion of atoms was accelerated with the increase in temperature, promoting the enhancement of densification. On the other hand, when the sintering temperature reached 460 K, the amorphous powders were situated in the supercooled liquid-phase transition region, where the samples were more susceptible to viscous flow, which would further promote rapid and highly densification of the samples. However, when the temperature was raised to 480 K~500 K, the density increased more slowly, which probably resulted from the fact that the glass samples precipitated crystalline phases and generated coarse grains under the high temperature, causing the viscous flow and the atomic motion to be hindered, thereby slowing down the increasing degree of densification.

**Figure 5:**
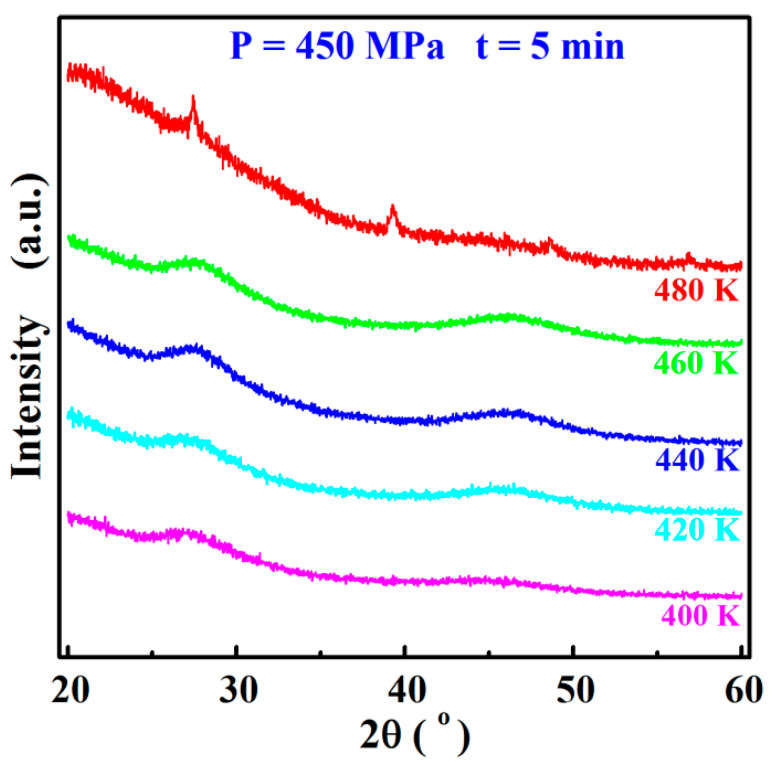
XRD patterns of Sn8 samples with different sintering temperature.

To clarify the influences of sintering temperature upon the microstructure within the materials, the sintered Sn8 samples were analyzed by XRD measurements, and the results are shown in Figure 5. When the sintering temperature was in the range of 400 K~460 K, only two broadened diffuse scattering peaks were observed in the XRD patterns of all the sintered samples, and no crystalline characteristic diffraction peaks were detected, demonstrating that as the sintering temperature was lower than 460 K, the sintered bulk samples still maintained the completely glassy structure. 

Once the sintering temperature was 480 K, multiple crystal characteristic peaks were identified in the XRD patterns of the sintered samples, indicating that the samples were no longer completely amorphous during the sintering process but rather crystallized, which also verified the correctness of the assumption in the previous paper that the slowly increasing density caused by the high sintering temperature might be attributed to the precipitation of the crystalline phases.

**Figure 6:**
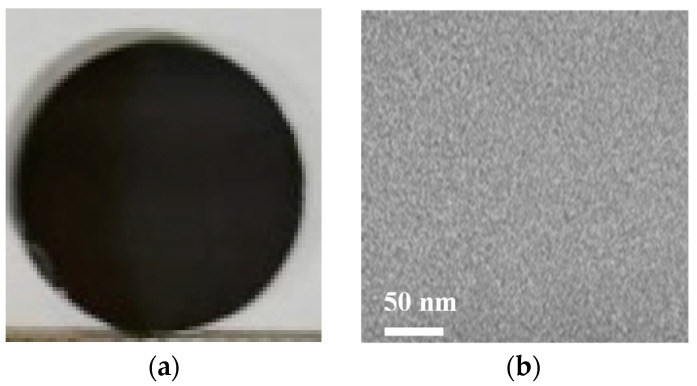
Morphology (**a**) and TEM image (**b**) of Sn8 bulk glass after spark plasma sintering.

The morphology and TEM image of the Sn8 bulk sample after spark plasma sintering are shown in Figure 6. A clean and uniform grayish matrix morphology without any diffusely distributed can be observed in the TEM bright-field image as shown in Figure 6b, reaffirming the completely glassy structure of the Sn8 sample.

In summary, the high-density (>96%) Sn8 bulk glass with the density of 5.5917 g/cm^3^ was successfully prepared by utilizing the SPS technique at 460 K, using a 5 min dwell time and 450 MPa pressure.

### 3.2. Electrical and Thermal Transport Properties

**Figure 7:**
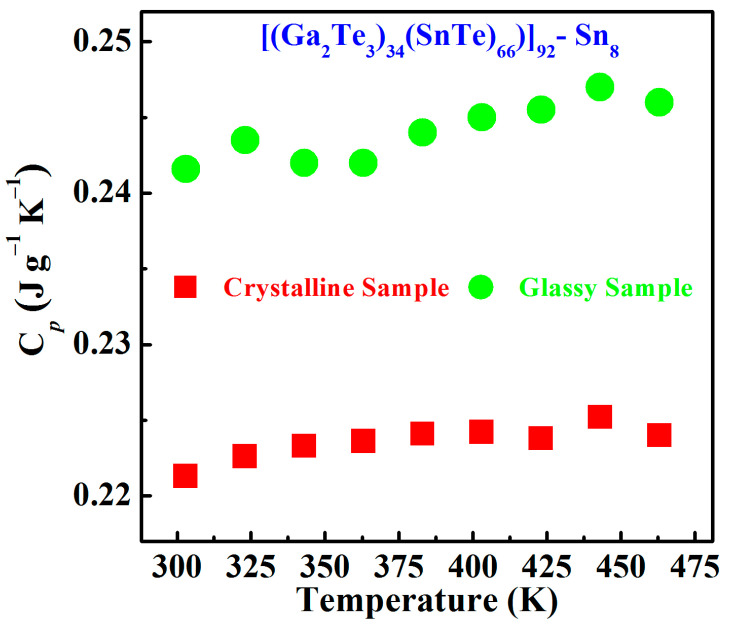
The temperature dependence of the specific heat *C_p_* for Sn8 bulk samples.

Figure 7 exhibits the temperature dependence of the specific heat *C_p_* for Sn8 bulk crystalline and glassy samples. Only slight fluctuations in the specific heat values of the two sintered samples were observed as the temperature increased, rather than the significantly increasing changes. This was because the maximum measured temperatures of the samples (463 K) were lower than the glass transition temperatures of the glassy samples (*T_g_* = 488 K) and the phase transition temperatures of the crystalline samples (*T_m_* = 930 K). 

Furthermore, the average specific heat capacity of the glassy Sn8 samples (*C_p-glass_* ≈ 0.24 J∙g^−1^∙K^−1^) is slightly higher than that of the crystalline ingot samples (*C_p-crystal_* ≈ 0.22 J∙g^−1^∙K^−1^), over the temperature range of 303~463 K. An explanation for this experimental phenomenon could be accounted for by the fact that, compared to the regular arrangement of atoms and strong inter-molecular forces of the crystalline state, the atomic structure of the glassy materials exhibited the disordered and chaotic arrangement with more voids and inter-facial energies, which resulted in weaker inter-molecular interactions and impeded inter-molecular thermal motion, and consequently, the specific heat of the glassy materials was generally slightly larger than that of the crystalline samples with the same chemical compositions [32,33].

**Figure 8:**
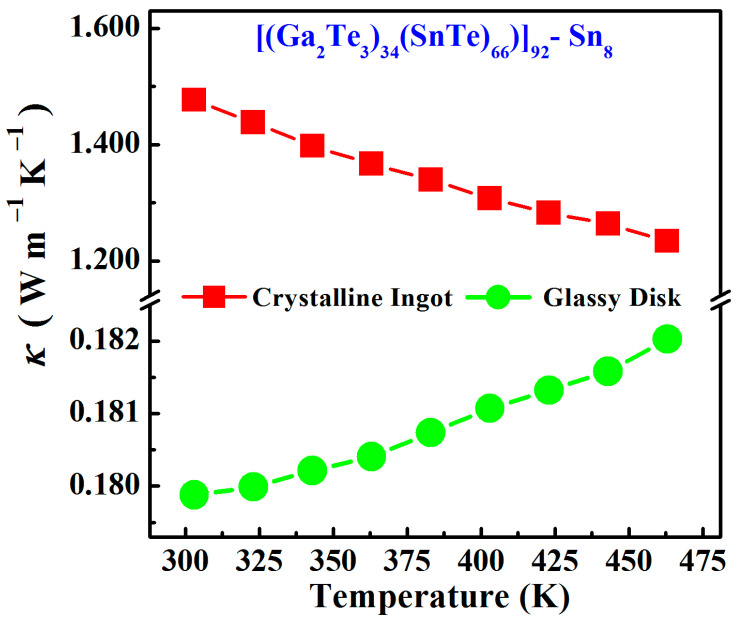
Thermal conductivity as functions of temperature for Sn8 bulk samples.

The thermal conductivity as a function of temperature for Sn8 bulk samples is presented in Figure 8. For the crystalline ingot samples, the thermal conductivity decreased from 1.476 W m^−1^∙K^−1^ at 303 K to 1.237 W m^−1^∙K^−1^ at 463 K with increasing measurement temperature, originating from the fact that higher temperatures enhanced the phonon scattering effects in crystalline samples, suppressed the thermal diffusive motion of the atoms, and resulted in the decrease in thermal conductivity. 

However, the glassy samples showed the opposite trend, that is, the thermal conductivity of the Sn8 glassy samples increased with increasing temperature. In glassy materials, the atoms were disorganized, and the thermal transports were no longer phonon diffusion but atomic/molecular vibrations [34]. Therefore, as the temperature increased, the molecular vibrations intensified and the thermal diffusion coefficient enhanced, causing an increase in the thermal conductivity. Notably, the Sn8 glass presented extremely low thermal conductivity values (0.179~0.182 W m^−1^∙K^−1^) in the measured temperature range from 303 K to 463 K, far lower than their crystalline ingot sample (~1.353 W m^−1^∙K^−1^), arising from the highly disordered structural of glassy materials. Compared to the conventional As-Te-based and Ge-Te-based glasses [35,36], the Sn8 glass studied in this paper still presented the lower thermal conductivity, since for most of the S-based, Se-based, and Te-based chalcogenide glassy materials, the thermal conductivity is generally negatively correlated with their molecular weights [37,38].

**Figure 9:**
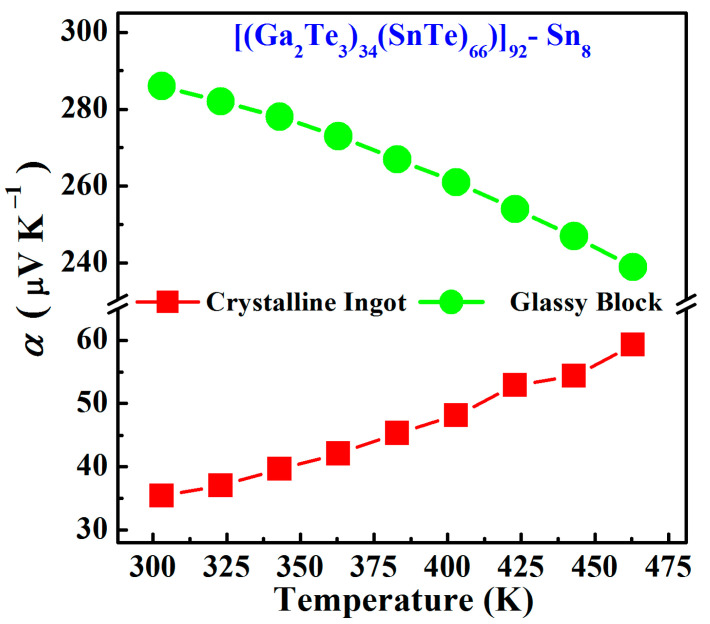
Seebeck coefficient as functions of temperature for Sn8 bulk samples.

As shown in Figure 9, the positive Seebeck coefficient values for all samples demonstrate that the samples belong to the P-type conductivity during the electrical transport; that is, the hole concentrations for the Sn8 samples were much larger than those of the free electrons, mainly depending on the hole conductivity. 

For Sn8 glassy samples, the Seebeck coefficient decreased with increasing temperature from 286 μV∙K^−1^ at 303 K to 239 μV∙K^−1^ at 463 K. Whereas the crystalline samples exhibited the reverse trend by increasing from 35 μV∙K^−1^ to 59 μV∙K^−1^ as the temperature increased. This is because the increased temperature depressed the intrinsic excitation of the crystalline samples with higher hole concentrations and promoted the excitation of the glassy samples with low hole concentrations [35]. Moreover, the Sn8 crystalline samples have shown low Seebeck values (<60 μV∙K^−1^) over the whole measurement ranges, whereas the Sn8 galssy samples exhibited a significantly optimized Seebeck coefficient, which increased up to 280 μV∙K^−1^. Generally, the higher the Seebeck coefficient, the larger the thermoelectric efficiency, and thus, the Sn8 glassy samples reveal the better thermoelectric potential.

**Figure 10:**
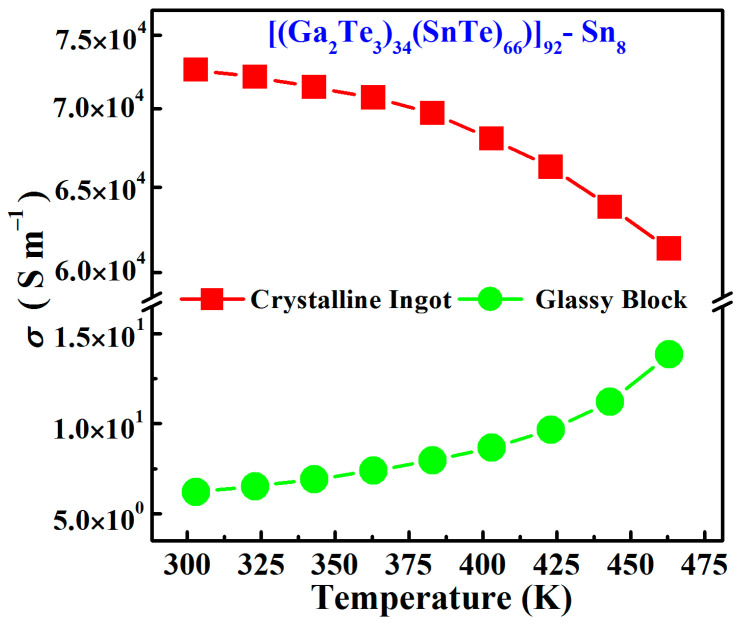
Electrical conductivity as functions of temperature for Sn8 bulk samples.

Figure 10 illustrates the temperature dependence of the electrical conductivity for Sn8 bulk samples. In the case of Sn8 crystalline samples, the electrical conductivity decreased with increasing temperature, exhibiting semi-metallic conducting features. Conversely, the electrical conductivity of Sn8 bulk glass increased with increasing temperature (from 6.2 S∙m^−1^ at 303 K to 13.8 S∙m^−1^ at 463 K), revealing typical semiconducting transport characteristics. Compared to the high room-temperature electrical conductivity value of Sn8 crystalline material (7.2 × 10^4^ S∙m^−1^), the room-temperature electrical conductivity of Sn8 glassy sample decreased by four orders of magnitude, and such change was mainly caused by the high degree of disordered structure, which induced the increment of the energy bands and the enhancement of the electron scattering, and hence, the electrical transports were hindered and presented a lower electrical conductivity. 

Generally, for glassy materials, the higher the glass transition temperature, the larger the maximum operating temperature, and the better the thermoelectric property. Reassuringly, the newly fabricated Sn8 bulk glass in this paper presented a high room-temperature conductivity (*σ =* 6.2 S∙m^−1^) and a large glass transition temperature (*T_g_ =* 488 K), exceeding the values of more than 1000-fold for the electrical conductivity and 50 K for the glass transition temperature compared to the typical undoped As_55_Te_45_ and Ge_20_Te_80_ glassy samples [35,39]. Meanwhile, compared to the Cu-Ge-Te-based [36], Cu-As-Te-based [15,40,41], Cu/Ag-As-Se-Te-based [42,43,44], and Ge-As-Te-based [35] glassy systems with low doping concentration (<10%), the Sn8 bulk glass still exhibited a favorable electrical conductivity and glass transition temperature, emerging as promising TE materials. This is because the Ga_2_Te_3_–SnTe system featured a much narrower band-gap and large molecular weight than the conventional tellurium-based Ge-Te or As-Te glassy systems, thereby exhibiting higher electrical conductivity and lower thermal conductivity. Whereas, the doping of the highly conductive metal Sn further enhanced the electrical conductivity of the glassy system, resulting in higher room-temperature conductivity for the Sn8 glass.

## 4. Conclusions

In this paper, the high-density Sn8 bulk samples were fabricated by utilizing the SPS technology, and the effects of sintering parameters on the density and microstructure were elucidated. Then, the thermal conductivity, Seebeck coefficient and electrical conductivity of Sn8 crystalline and glassy samples were systematically analyzed.

(1) The high-density (>96%) Sn8 bulk glass with the density of 5.5917 g/cm^3^ was successfully prepared by the SPS technology at 460 K, using a 5 min dwell time and 450 MPa pressure.
(2) For the Sn8 bulk materials, the room-temperature thermal conductivity significantly decreased from 1.476 W m^−1^∙K^−1^ in the crystalline sample to 0.179 W m^−1^∙K^−1^ in the glass, and the Seebeck coefficient obviously increased from 35 μV∙K^−1^ in to 286 μV∙K^−1^. Thus, the glass transition of tellurium-based semiconductors could significantly degrade the phonon energy and transmission rate, presenting lower thermal conductivities and higher Seebeck coefficients, which render tellurium-based glasses appear as newly emerging thermoelectric materials.
(3) Compared to the conventional tellurium-based glassy systems, the newly fabricated Sn8 bulk glass presented a high room-temperature conductivity (*σ =* 6.2 S∙m^−1^) and a large glass transition temperature (*T_g_ =* 488 K), which was expected to be a promising thermoelectric material.

## Data Availability

Data are contained within the article.
